# Association between sedentary behaviour and socioeconomic factors, diet and lifestyle among the Balearic Islands adolescents

**DOI:** 10.1186/1471-2458-12-718

**Published:** 2012-08-30

**Authors:** Maria del Mar Bibiloni, Jordi Pich, Alfredo Córdova, Antoni Pons, Josep A Tur

**Affiliations:** 1 Research Group on Community Nutrition and Oxidative Stress, University of Balearic Islands, Guillem Colom Bldg, Campus, E-07122 Palma de Mallorca, Spain; 2 Department of Physiology and Biochemistry, School of Health Sciences, University School of Physiotherapy, University of Valladolid, Soria, 42004, Spain

## Abstract

**Background:**

Many different factors influenced food habits and physical activity patterns of adolescents in a complex interactive way. The aim of this study was to assess association between sedentary behaviour and socioeconomic factors, diet and lifestyle among the Balearic Islands adolescents.

**Methods:**

A cross-sectional survey (*n* = 1961; 12–17 years old) was carried out. Physical activity was assessed using the International Physical Activity Questionnaire for adolescents (IPAQ-A). Sedentary behaviour was defined as <300 min/week of moderate and vigorous physical activity. Anthropometric measurements, body image, socio-economic and lifestyle determinants, food consumption, and adherence to the Mediterranean diet were assessed.

**Results:**

The prevalence of sedentary behaviour was 37.1% (22.0% boys, 50.8% girls). Active boys consumed frequently breakfast cereals and fresh fruit; active girls yogurt, cheese, breakfast cereals, and fresh fruit; and sedentary girls high fat foods and soft drinks. Sedentary behaviour of girls was directly associated to age, and time spent on media screen and homework, and inversely related to adherence to Mediterranean diet, and body composition. Sedentary behaviour of boys was inversely related to adherence to the Mediterranean diet, and the desire to remain the same weight.

**Conclusions:**

The prevalence of sedentary behaviour among Balearic Islands adolescents is high, mainly among girls. Age, sex, parental educational and profession levels, body size dissatisfaction, and poor quality diet are important factors of physical activity practice among adolescents.

## Background

Adolescence is a transitional stage and many changes take place at physiologic and behavioural levels, representing an important life stage for the development of healthy nutrition [1] and physical activity behaviours. Many different factors influenced food habits and physical activity patterns in a complex interactive way [2]. Socio-cultural factors as parental occupational status, maternal level of education, cultural and/or religious habits, and the role of family and patterns of beauty are factors that have a strong influence on eating habits [3] and physical activity in adolescents [4-7].

Reductions in physical activity in youth as a result of adopt a major inactive lifestyle, increasing time spent watching television, playing video games and Internet over the past two decades are believed to explain part of the rising prevalence of obesity in children and adolescents [8-10]. The prevalence of overweight and obesity among children and adolescents has risen greatly worldwide [8], with current estimates suggesting that around 30% of boys and 25% of girls in the Balearic Islands are overweight or obese [11]. However, physical inactivity has been related not only to obesity but also to the associated morbidity and non transmittable chronic diseases [12], being one of the major importance in public health because is highly prevalent [13,14].

Cohort studies have evidenced that physical inactivity during childhood and/or adolescence tends to continue into adulthood, becoming difficult to change [7,15]. However, among environmental factors of obesity and risk of disease the lack of regular physical activity is potentially modifiable [13] and programs to stimulate physical activities in this age range should be a priority for public health policies and a focus for teachers [7]. Therefore, the aim of this study was to assess association between sedentary behaviour and socioeconomic factors, diet and lifestyle among Balearic Islands adolescents.

## Methods

### Study design

The study is a population-based cross-sectional nutritional survey carried out (2007–2008) in the Balearic Islands (Spain), a Mediterranean region.

### Selection of participants, recruitment and approval

A multicenter study was performed on Balearic Islands’ adolescents aged 12–17 years. The population was selected by means of a multiple-step, simple random sampling, taking into account first the location (Palma de Mallorca, Calvià, Inca, Manacor, Maó, Eivissa, Llucmajor, Santa Margalida, S’Arenal, Sant Jordi de Ses Salines) and then by random assignment of the schools within each city. Sample size was stratified by age and sex. The socio-economic variable was considered to be associated to geographical location and type of school. As the selection of schools was done by random selection and fulfilling quota, this variable was also considered to be randomly assigned.

To calculate the number of adolescents to be included in the study in order to guarantee a representative sample of the whole Balearic Islands, we selected the variable with the greatest variance for this age group from the data published in the literature at the time the study was planned; that was BMI [16]. The sampling was determined for the distribution of this variable; the CI was established at 95% with an error ± 0.25. The established number of subjects was 2400. The total number of subjects was uniformly distributed in the cities and proportionally distributed by sex and age group. Exclusion criteria were: type 2 diabetes, pregnancy, alcohol or drug abuse, and non-directly related nutritional medical conditions.

The sample was oversized to prevent loss of information and as necessary to do the fieldwork in complete classrooms. In each school, classrooms were randomly selected among those of the same grade or level, and all the adolescents of one classroom were proposed to participate in the survey. A letter about the nature and purpose of the study informed parents or legal tutors. After receiving their written consent, the adolescents were considered for inclusion in the study. All responses of questionnaires were filled in by adolescents. After finishing the field study, the adolescents who did not fulfil the inclusion criteria were excluded. Finally, the sample was adjusted by a weight factor in order to balance the sample in accordance to the distribution of the Balearic Islands’ population and to guarantee the representativeness of each of the groups, already defined by the previously mentioned factors (age and sex). The final number of subjects included in the study was 1961 adolescents (82% participation). The reasons to not participate were (a) the subject declined to be interviewed, and (b) the parents did not authorize the interview.

This study was conducted according to the guidelines laid down in the Declaration of Helsinki, and all procedures involving human subjects were approved by the Balearic Islands’ Ethics Committee (Palma de Mallorca, Spain).

### Anthropometry measurements

Height was determined using a mobile anthropometer (Kawe 44444, Asperg, Germany) to the nearest millimetre, with the subject’s head in the Frankfurt plane. Body weight was determined to the nearest 100 g using a digital scale (Tefal, sc9210, Rumilly, France). The subjects were weighed in bare feet and light underwear. Waist circumference (WC) and hip circumference (HC) were measured using a non-stretchable measuring tape (Kawe, 43972, Asperg, Germany). The subjects were asked to stand erect in a relaxed position with both feet together on a flat surface. WC was measured as the smallest horizontal girth between the costal margins and the iliac crests at minimal respiration. Measurements were made to the nearest 0.1 cm. HC was taken as the greatest circumference at the level of greater trochanters (the widest portion of the hip) on both sides. Measurements were made to the nearest 0.1 cm. Triceps and subscapular skinfold thickness (ST) were measured at the right side of the using a Holtain skinfold caliper (Tanner/Whitehouse, Crosswell, Crymych, UK), and the mean of three measurements was used. Body fat percentage (%BF) was measured from triceps and subscapular ST according to Slaughter et al. [17]. This equation has been proposed as the most accurate for estimation of %BF from ST in this particular population of adolescents [18]. Height and weight measures were used to calculate body mass index (BMI, kg/m^2^) and WC and height were used to calculate waist-to-height ratio (WHtR). %BF and height were used to calculate fat mass index (FMI; kg/m^2^).

### Defining overweight and obesity

Adolescents were classified into three groups as follows: (i) not at risk (BMI for age and sex < P97, and FMI < 4.58 kg/m^2^ in boys, FMI < 7.76 kg/m^2^ in girls); (ii) overweight (BMI for age and sex < P97, and FMI ≥ 4.58 kg/m^2^ in boys, FMI ≥ 7.76 kg/m^2^ in girls); and (iii) obesity (BMI for age and sex ≥ P97, and FMI ≥ 4.58 kg/m^2^ in boys, FMI ≥ 7.76 kg/m^2^ in girls). The variable was labeled as ‘body composition’ [19]. Age- and sex-specific BMI cut-offs were used according to the International Obesity Task Force and Cole et al. definitions [20], and FMI cut-offs according to Alvero-Cruz et al. [21] criteria for adolescents: 4.58 kg/m^2^ in boys and 7.76 kg/m^2^ in girls, as the limit between normal-fat and overfat.

### Body image

Perceived body image was measured using the Stunkard scale [22], which consists of silhouette drawings ranging from 1 to 9 with monotonic increments in overweight percentage where 1 is the leanest and 9 is the heaviest. Separate figures for boys and girls were used. Participants were asked to identify of the 9 body figures: (a) ‘Which silhouette looks most like yourself?’ and (b) ‘Which silhouette would you like to look like?’ The difference between perceived body image and desired body image was used to determine the level of dissatisfaction with current body image. Values other than zero represent dissatisfaction with perceived body image. A positive value was indicative of the participant’s desire to be thinner than his/her perceived current size, while a negative value reflected the participant’s desire to be thicker than his/her current perceived size [23,24].

### Assessment of physical activity

Physical activity was evaluated according to the guidelines for data processing and analysis of the international physical activity questionnaire (IPAQ) [25] in the short form, and its specific modification for adolescents (IPAQ-A) [26]. The specific types of activity assessed were walking, and moderate (i.e. physical activity at school), and vigorous (i.e. sport practice) activity. According to the AVENA (Food and Assessment of Nutritional Status of Adolescents) study [27], the questionnaire also included information on television (TV) viewing, computer use and video games and homework in h/d, and usual sleep duration to the nearest 10 min. According to recent reports of physical activity for adolescents [7,28], sedentary behaviour was established with a cut-off level of 300 min of moderate/vigorous physical activity (MVPA) per week.

### Socioeconomic factors

Socio-demographic factors were recorded using a questionnaire that included age group; parental education level (according to years and type of education: low, <6 years; medium, 6–12 years; high, >12 years); and parental profession level (based on the occupation of parents and classified as low, medium and high, according to the Spanish Society of Epidemiology [29]. The number of daily meals and snacks was calculated from the total eating occasions that participants declared among the following: breakfast; mid-morning snack; lunch; mid-afternoon snack; dinner; before going to sleep; others. According to previous studies that found an inverse relationship between eating frequency and BMI [11], three groups of eating frequency were considered: ≤3, 4 and ≥5 times/d.

### Dietary assessment

A semi-quantitative food-frequency questionnaire (FFQ) previously validated [30] was used. The FFQ, which asked the subject to recall average use over the past year, consisted of 145 items (118 of the original validated FFQ plus the most characteristic Balearic Islands foods in order to make easy the interviewee answer), and arranged by food type and meal pattern. Edible fractions of foods were recorded in the database. Frequency of food consumption was based on times that food items were consumed (per day, week or month). Consumption <1/month was considered no consumption. The period of consumption of seasonal items was also considered. The 145 foods items from the FFQ were collapsed to twenty-nine food groups, which may have practical importance in daily diet and clinical practice among Mediterranean younger [31,32].

### Adherence to the Mediterranean diet

The degree of adherence to the Mediterranean diet was evaluated using the KIDMED index (Mediterranean diet quality index for children and adolescents), described elsewhere [33]. Based on the given answers, the test classified individuals according to the quality of the Mediterranean diet categorized as: high, medium or poor.

### Statistics

Analyses were performed with Statistical Package for the Social Sciences version 19.0 (SPSS, Inc., Chicago, IL, USA). All tests were stratified by sex. Significant differences in prevalence were calculated by means of *χ*^2^. Differences between means were tested using ANOVA. Logistic regression models with the calculations of corresponding adjusted odds ratios and 95% confidence intervals were used to examine possible differences between those adolescents who were sedentary or active. Univariate analysis was first carried out for all the socio-demographic and lifestyle variables that could be associated with sedentary behaviour. Any factor that was significantly associated was considered as a candidate for the multivariate model. Multiple logistic regression analyses were used to simultaneously examine the effect of different socio-demographic and lifestyle variables on the prevalence of sedentary behaviour. Multiple logistic regression analyses were also used to simultaneously examine the association between sedentary behaviour and dietary patterns adjusted by potential confounders (e.g. age, parental education level, and parental profession level, body composition, number of daily meals and snacks, desire for weight change, and energy intake). Level of significance for acceptance was *P* < 0.05.

## Results

Table 1 shows physical activity and lifestyle of Balearic Islands adolescents stratified by sex and age. Sedentary behaviour among adolescents was 37.1%. Boys were more active and spend more weekly time in both moderate and vigorous physical activity than girls, and boys were devoted more weekly time to vigorous physical activity (64%) than girls (50%). Girls (50.8%) showed higher sedentary behaviour than boys (22.0%), and this prevalence increased with age, whereas time devoted to vigorous physical activity decreased with age; the physical activity practice decreases annually 1.3% (boys) and 3.2% (girls). Time devoted to media screen was higher among 14-y.o. boys and girls; and time spent on homework, and sleep time increased with age in girls, but not in boys.

**Table 1:** Physical activity and lifestyle of Balearic Islands adolescents stratified by sex and age

		**Age group**				
		**Total**	**12-13**	**14-15**	**16-17**	***P***
**Boys**	***n***	939	240	445	254	
	**Physical activity**^**1**^**(%)†**					
	Inactive	22.0	17.6	23.2	24.0	NS
	Active	78.0	82.4	76.8	76.0	
	**Physical activity practice**					
	**Moderate**					
	Did not engage (%)†	8.9	7.2	7.6	12.8	*
	Mean duration (min/week)‡	215.1 ± 250.5	264.9 ± 330.2^a^	192.6 ± 200.7^a^	208.4 ± 237.7	**
	Time spent (% total)†	35.7 ± 28.6	35.2 ± 24.6	35.9 ± 27.3	35.7 ± 28.6	NS
	**Vigorous**					
	Did not engage (%)†	11.0	8.0	11.1	13.6	NS
	Mean duration (min/week)‡	502.8 ± 401.4	558.7 ± 454.2	486.6 ± 387.3	477.8 ± 367.6	NS
	Time spent (%)†	64.3 ± 27.0	64.8 ± 24.6	64.1 ± 27.3	64.3 ± 28.6	NS
	**Media-screen time (%)†**					
	<2 h/d	17.6	21.2	16.0	17.1	*
	2-4 h/d	42.6	47.7	41.1	40.2	
	≥4 h/d	39.8	31.1	42.8	42.7	
	**Homework time (%)†**					
	<1 h/d	16.3	13.8	19.1	13.8	NS
	1-3 h/d	69.7	69.7	69.3	70.4	
	≥3 h/d	14.0	16.5	11.6	15.8	
	**Sleep time (%)†**					
	<7 h/d	9.5	7.1	9.2	12.2	NS
	≥7 h/d	90.5	92.9	90.8	87.8	
**Girls**	***n***	1022	255	503	264	
	**Physical activity**^**1**^**(%)†**					
	Inactive	50.8	41.4	51.9	57.3	**
	Active	49.2	58.6	48.1	42.7	
	**Physical activity practice**					
	**Moderate**					
	Did not engage (%)†	12.7	7.9	11.0	20.0	***
	Mean duration (min/week)‡	169.1 ± 165.7	188.5 ± 210.5	164.1 ± 147.1	158.8 ± 146.1	*
	Time spent (% total)†	50.4 ± 33.7	47.2 ± 31.6	52.0 ± 33.7	50.6 ± 35.8	NS
	**Vigorous**					
	Did not engage (%)†	28.4	20.3	29.4	33.8	**
	Mean duration (min/week)‡	354.3 ± 356.2	422.6 ± 470.4^a,b^	338.9 ± 313.8^a^	308.5 ± 267.2^b^	**
	Time spent (%)†	49.6 ± 33.7	52.8 ± 31.6	48.0 ± 33.7	49.4 ± 35.8	NS
	**Media-screen time (%)†**					
	<2 h/d	21.2	28.0	17.5	21.6	**
	2-4 h/d	38.7	41.0	36.3	40.9	
	≥4 h/d	40.2	31.0	46.2	37.5	
	**Homework time (%)†**					
	<1 h/d	8.3	8.9	10.0	4.3	**
	1-3 h/d	66.0	68.5	67.6	60.9	
	≥3 h/d	25.7	22.6	22.4	34.8	
	**Sleep time (%)†**					
	<7 h/d	8.9	3.1	9.7	12.9	***
	≥7 h/d	91.1	96.9	90.3	87.1	

^1^Physical inactivity was defined as <300 min/week of moderate and vigorous physical activity [7]. Values are mean ± SD and%. Significant differences between age groups by: †*χ*^2^ and ‡ANOVA: *p < 0.05; **p < 0.01; ***p < 0.001. NS: not significant. Significant differences between pairs of means by Bonferroni’s *post-hoc* test (*P* < 0.05): ^a^‘12-13’ vs. ‘14-15’; ^b^‘12-13’ vs. ‘16-17’.

Table 2 shows anthropometric characteristics of Balearic Islands adolescents according to physical activity practice and stratified by sex. Sedentary boys showed higher adiposity (measured by means of BMI, WC, HC, TSF, SCSF,%BF and WHtR), and sedentary girls showed lower weight, BMI and HC than their active counterparts.

**Table 2:** Anthropometric characteristics of Balearic Islands adolescents, according to physical activity practice and stratified by sex

	**Boys**			**Girls**		
	**Sedentary adolescents**	**Active adolescents**	***P***	**Sedentary adolescents**	**Active adolescents**	***P***
***n***	207	732		519	503	
**%**	22.0	78.0		50.8	49.2	
Weight (kg)	65.1 ± 13.1	63.2 ± 13.0	NS	56.1 ± 11.0	57.8 ± 11.1	*
Height (cm)	169.5 ± 8.6	170.3 ± 8.7	NS	161.0 ± 6.6	160.8 ± 6.6	NS
BMI (kg/m^2^)	22.5 ± 3.8	21.7 ± 3.8	**	21.6 ± 4.0	22.3 ± 3.8	**
WC (cm)	74.7 ± 8.6	72.9 ± 8.4	*	67.8 ± 8.2	68.6 ± 8.1	NS
HC (cm)	93.6 ± 9.9	91.6 ± 10.0	*	93.5 ± 9.9	95.0 ± 9.8	*
TSCF (mm)	11.7 ± 5.1	10.5 ± 5.1	**	15.3 ± 5.3	15.2 ± 5.3	NS
SCSF (mm)	12.1 ± 3.2	10.1 ± 5.9	***	12.9 ± 6.0	13.1 ± 6.0	NS
BF (%)	19.2 ± 4.6	16.5 ± 8.4	***	24.2 ± 6.4	24.1 ± 6.4	NS
FMI	4.7 ± 2.8	3.8 ± 2.8	***	5.4 ± 2.2	5.6 ± 2.3	NS
WHtR	0.5 ± 0.06	0.4 ± 0.05	**	0.4 ± 0.04	0.4 ± 0.04	NS

Abbreviations: BMI, body mass index; WC, waist circumference; HC, hip circumference; TSCF, tricipital skinfold thickness; SCSF, subscapular skinfold thickness; BF, body fat; FMI, fat mass index; WHtR, waist-to-height ratio.

Values are mean ± SD. Significant differences between sedentary and active adolescents by ANCOVA adjusted for age, parental profession level, parental education level, number of daily meals & snacks, and adherence to the Mediterranean diet.

*p < 0.05; **p < 0.01; ***p < 0.001. NS: not significant.

Food consumption among active and sedentary Balearic Islands adolescents is showed in Table 3. Active boys consumed frequently breakfast cereals and fresh fruit than their sedentary peers. Active girls consumed frequently yogurt and cheese, breakfast cereals, and fresh fruit than their sedentary counterparts. Sedentary girls consumed more frequently high fat foods and soft drinks than their active peers.

**Table 3:** Food consumption among active and sedentary Balearic Islands adolescents

**Food groups**	**Frequency consumption**	**Boys**			**Girls**		
		**Active adolescents (%)**	**Sedentary adolescents (%)**	***P***	**Active adolescents (%)**	**Sedentary adolescents (%)**	***P***
		**n=732**	**n=207**		**n=519**	**n=503**	
**Dairy products**							
Milk†	≥7 times/week	77.2	76.8	0.906	67.6	64.5	0.333
Yogurt & cheese	≥7 times/week	68.4	62.4	0.123	64.5	56.1	0.009
Dairy desserts	≥2 times/week	71.5	72.2	0.863	61.9	62.9	0.763
**Meat**							
Red meat†	≥2 times/week	53.0	55.9	0.489	39.6	42.8	0.314
Poultry & rabbit†	≥2 times/week	15.6	14.4	0.698	17.7	21.3	0.171
Sausages†	≥5 times/week	54.9	56.2	0.754	46.5	48.1	0.644
	2-4 times/week	25.8	25.4	0.922	26.2	21.3	0.086
	≤4 times/month	19.3	18.4	0.774	27.3	30.6	0.271
**Fish & seafood**†	≥2 times/week	20.1	14.5	0.089	19.3	14.8	0.071
**Eggs**†	≥2 times/week	31.4	38.7	0.062	23.8	22.2	0.558
**Legumes**	≥2 times/week	20.8	19.8	0.772	19.3	18.3	0.685
**Cereals, grains & products**							
Bread†	≥7 times/week	86.5	82.8	0.201	82.9	81.1	0.475
Breakfast cereals†	≥5 times/week	47.1	36.6	0.011	34.7	26.4	0.006
Biscuits	≥5 times/week	51.3	53.5	0.592	49.1	44.2	0.139
Pasta†	≥5 times/week	8.7	8.0	0.778	6.6	5.8	0.603
Rice dishes†	≥5 times/week	8.7	9.1	0.860	6.6	7.0	0.789
Pizza†	≥2 times/week	19.9	14.4	0.093	11.4	12.8	0.525
**Fruits**							
Fresh fruit†	≥2/day	30.4	21.9	0.024	40.2	22.5	0.000
Fruit juices	≥7 times/week	54.0	46.5	0.074	52.3	45.7	0.046
Canned fruit	≥2 times/week	7.2	7.5	0.878	4.9	3.0	0.141
**Vegetables**†	≥2/day	8.2	7.5	0.748	11.5	13.2	0.415
**Nuts**†	≥2 times/week	36.2	31.0	0.193	23.3	25.1	0.533
**Potatoes & tubercles**†	≥2 times/week	42.0	44.9	0.471	32.1	32.1	0.990
**Fats**							
Olive oil	≥7 times/week	52.5	45.7	0.104	57.0	55.0	0.530
Others oils & fats	≥2 times/week	36.8	34.4	0.556	36.3	37.0	0.829
High fat foods	≥5 times/week	41.9	42.5	0.870	26.8	36.8	0.001
	2-4 times/week	29.0	23.8	0.167	29.0	29.4	0.905
	≤4 times/month	29.2	33.7	0.241	44.2	33.8	0.001
**Drinks**							
Soft drinks	≥5 times/week	55.4	55.4	0.986	32.0	44.6	0.000
	2-4 times/week	12.2	9.1	0.254	10.1	10.9	0.691
	≤4 times/month	32.4	35.5	0.429	57.9	44.4	0.000
Tea & coffee	≥2 times/week	15.1	12.4	0.363	14.2	18.7	0.069
Alcoholic beverages	≥1 times/week	11.1	9.6	0.561	4.2	5.4	0.427
**Other foods**							
Sweets	≥5 times/week	69.9	70.7	0.830	77.8	74.6	0.264
	2-4 times/week	16.4	13.8	0.408	12.8	16.2	0.152
	≤4 times/month	13.7	15.5	0.559	9.4	9.2	0.921
Chocolates	≥5 times/week	17.0	21.5	0.166	16.7	16.1	0.791
	2-4 times/week	12.2	13.3	0.697	10.9	12.2	0.537
	≤4 times/month	70.8	65.2	0.151	72.4	71.7	0.825

†Food consumption cut-offs [31,32]. Significant differences between active and sedentary adolescents by *χ*^2^ test.

Association between sedentary behaviour and socioeconomic factors, diet and lifestyle among the boys and girls are showed in Table 4. Univariate analysis showed that parental educational and profession levels are directly associated to sedentary behaviour in girls, and body composition to sedentary behaviour in boys (data not shown). Multivariate analysis showed that sedentary behaviour of girls was directly related to age, and time spent on media screen and homework, and inversely related to adherence to Mediterranean diet, and body composition, and that sedentary behaviour of boys was inversely related to adherence to the Mediterranean diet, and the desire to remain the same weight.

**Table 4:** Association between sedentary behaviour and socioeconomic factors, diet and lifestyle among Balearic Islands adolescents

**Variable**	**Boys**		**Girls**	
	**Sedentary adolescents (%)**	**Adjusted OR (95% CI)**	**Sedentary adolescents**	**Adjusted OR**
**Age group**				
12-13 years old	17.6	0.70 (0.39-1.23)	41.1	0.61 (0.40-0.95)*
14-15 years old	23.2	0.90 (0.58-1.41)	51.9	0.91 (0.64-1.29)
16-17 years old	24.0	1.00 (ref.)	57.3	1.00 (ref.)
**Parental education level**				
Low	22.9	0.85 (0.50-1.44)	56.1	1.18 (0.76-1.82)
Medium	21.4	1.21 (0.72-2.02)	51.0	1.18 (0.78-1.78)
High	20.2	1.00 (ref.)	43.5	1.00 (ref.)
**Parental profession level**				
Low	21.7	0.73 (0.37-1.42)	56.8	1.23 (0.74-2.06)
Medium	22.4	0.94 (0.53-1.69)	46.9	1.06 (0.67-1.69)
High	20.5	1.00 (ref.)	43.6	1.00 (ref.)
**Body composition**				
Not at risk	19.1	0.61 (0.28-1.36)	51.6	2.06 (1.03-4.13)*
Overweight	30.9	0.96 (0.45-2.04)	59.8	2.91 (1.29-6.57)*
Obesity	37.5	1.00 (ref.)	37.0	1.00 (ref.)
**Sleep**				
<7 h/d	26.2	0.97 (0.49-1.92)	50.0	0.88 (0.53-1.48)
≥7 h/d	21.6	1.00 (ref.)	50.8	1.00 (ref.)
**Media-screen time**				
<2 h/d	19.0	0.83 (0.47-1.48)	36.8	0.64 (0.42-0.99)*
2-4 h/d	20.9	0.92 (0.60-1.42)	50.1	0.94 (0.67-1.31)*
≥4 h/d	23.6	1.00 (ref.)	58.7	1.00 (ref.)
**Homework time**				
<1 h/d	28.1	1.67 (0.83-3.37)	45.5	0.46 (0.25-0.86)*
1-3 h/d	20.3	1.12 (0.62-2.02)	48.8	0.66 (0.47-0.94)*
≥3 h/d	22.5	1.00 (ref.)	57.1	1.00 (ref.)
**Number of daily meals & snacks**				
≤3	25.5	0.77 (0.46-1.28)	50.9	1.08 (0.73-1.61)
4	20.6	0.70 (0.44-1.12)	52.7	1.14 (0.77-1.70)
≥5	20.2	1.00 (ref.)	48.2	1.00 (ref.)
**KIDMED index**				
Poor –quality diet	25.5	1.77 (1.08-2.91)*	67.3	3.08 (1.83-5.17)***
Average –quality diet	24.5	0.97 (0.58-1.63)	52.2	1.57 (1.10-2.23)*
Good –quality diet	17.8	1.00 (ref.)	39.2	1.00 (ref.)
**Desire for weight change**				
To be thinner	31.3	0.23 (0.07-1.24)	50.7	0.77 (0.40-1.45)
To remain the same weight	13.0	0.47 (0.28-0.79)**	50.1	0.80 (0.42-1.52)
To be thicker	23.0	1.00 (ref.)	53.7	1.00 (ref.)

Abbreviations: *OR* odds ratio, *CI*, confidence interval, *ref* reference, *KIDMED*, Mediterranean Diet Quality Index; Adjusted OR: Multivariate analysis (multiple logistic regression analysis considering the simultaneous effect of all explanatory variables: age, parental education level, and parental profession level, body composition, number of daily meals and snacks, desire for weight change, and energy intake).*p < 0.05; **p < 0.01; ***p < 0.001.

## Discussion

Our study has assessed that sedentary behaviour among Balearic Islands adolescents is high (37.1%), mainly among girls (22% boys and 50.8% girls), but lower than those of American (55.9%) [34], Brazilian (56.9%) [35] and other European adolescents [36].

In our study boys were more active than girls in all age groups, and sex difference was accentuated with age for a significant declined physical activity practice in girls. Adolescence is the beginning of the decline of physical activity practice [37], which decreases annually about 2.7% among boys and 7.4% among girls between the ages of 10 and 17 [38]. Among Balearic Islands adolescents, the physical activity practice decreases annually, but less than among other adolescents. These results confirm that age and sex are important determinants of physical activity practice among adolescent population [39].

Parental educational and profession levels have been also associated with adolescents’ sedentary behaviour [7,39,40]. In our study, the intergenerational association between parental educational and professional levels with sedentary behaviour was observed by univariate, but not multivariate, analysis in girls. Among Canary Islands’ adolescents [41], father’s regular physical activity in case of boys and mother’s one in case of girls was positively associated with the likelihood of being active. Moreover, family usually supports engagement to physical activity practice more on boys than girls [42]. It has been also found that the access to physical activity spaces, by means of a combination of outdoor and indoor facilities, has been positively associated with the likelihood of being active in girls [41]. The access to these facilities is strongly related to the parental educational and profession level, which may explain our results.

The measurement of sedentary behaviour is not a well-developed field. We used a cut-off of <300 min/week of moderate and vigorous physical activity to define sedentary behaviour in adolescents. Our results showed that sedentary behaviour was associated with spending time on use media-screen and doing homework in girls, which agrees previous findings about sex differences in sedentary behaviours [43,44]. Our results suggest a displacement of physical activity to sedentary pursuits in girls with age, whereas in boys the use of media-screen or homework would not necessarily affect the physical activity practice. These findings agree previous results that pointed out that the excess time accumulated in front of screens is negatively associated with MVPA in adolescents [41]. However, our study shows that time spends on media-screen and homework are useful to distinguish between active and sedentary girls.

In the present study, a significant relationship was found between body composition and physical activity practice but this association was difficult to explain for boys and girls. Thus, in agreement with previous findings reported in young people and adults [4,45,46], overweight and obese boys were more sedentary than their normal weight (not at risk) counterparts; whereas obese girls were often more active than their lean peers. Although we cannot exclude the possibility that obese girls increased their physical activity as a method to self-control body weight, it may be possible that obese girls over-reported their physical activity practice.

Being self-conscious about one’s body size may play a role in influencing a variety of health behaviours, such as physical activity practice; however, limited research to date has been focused on the relationship of body size satisfaction and physical activity practice. Findings from the literature indicate gender differences between men and women in how they see their bodies and how this may affect their physical activity practice [47]. A previous study on adults suggested that both men and women who were unsatisfied with their body size were less likely to be regularly active than those who were satisfied^47^. Similar results in boys but not in girls were observed in our study, which could be explained because boys are more likely to practice physical activity as strategy to increase body weight and muscle mass [48], while girls are more likely to meet the patterns of beauty attempting to lose weight, mainly by diets [49]. Our findings also support the suggestion [47] that a sedentary lifestyle in unsatisfied boys may also lead a self-perpetuating vicious circle of low activity.

Sedentary behaviour has been associated with food choice, and cereals, fruits and vegetables often appear in the diet of active adults and children [50]. Children who follow a healthy diet are those who might also maintain high levels of physical activity [51]. In a previous study, we found that sedentary adolescents showed the lowest adherence to the Mediterranean dietary pattern [19]. The present results showed that sedentary adolescents often consumed less cereals, and fresh fruit, whereas high fat foods appeared often in their diet.

Moreover, we have lately described two major dietary patterns in Balearic Islands adolescent population: the ‘Western’ and the ‘Mediterranean’ pattern [19]. In that study we observed that adolescents who spent ≥4 h per day on use media-screen showed higher mean intake for most of the food categories included in the ‘Western’ dietary pattern (e.g. dairy desserts, red meat, sausages, bread, rice dishes, fruit juices, soft drinks, high-fat foods, sweets and chocolates), whereas mean intake for yogurt & cheese, fruit and vegetables was lower in them. Therefore, in addition to the promotion of physical activity, reductions in sedentary behaviour (i.e. media-screen time) with education programmes focused on strategies to promote healthy food choices (i.e.: following the Mediterranean diet) should be considered in nutrition education programmes for adolescents aimed at reducing risk of disease.

### Limitations of the study

In the literature there are important methodological differences in instruments and cut-off points which frequently prevent comparisons among studies [52]. Questionnaires have inherent limitations, mainly because they are subjective in nature. One bias could be that all responses of questionnaires were filled in by adolescents; however, doubts were solved immediately in the classroom by interviewers. Self-report of physical activity can lead to overreport the physical activity due to a social desirability bias, and therefore the number of inactive individuals may be lower than that reported [53,54], especially among children and adolescents, and also among obese [53]. Moreover, sedentary behaviours may be more difficult to remember than activities of higher intensity [55] and recall bias in self-reported sedentary behaviours have been reported among adolescents [56]. Therefore, objective methods are generally preferable in assessing dimensions of physical activity in young people [4]. An extensive range of instruments for measuring physical activity has been reported in the literature, but critical elements in the utility of an instrument to measure physical activity are to be relatively inexpensive, cause minimal inconvenience to the participant, and be able to be administered with relative ease [57]. However, in epidemiological studies, self-reporting is usually the most feasible method of assessing physical activity, especially when a high number of participants are interviewed [4].

## Conclusions

The prevalence of sedentary behaviour among Balearic Islands adolescents is high, mainly among girls. Age, sex, parental educational and profession levels, dissatisfaction with body size and poor quality diet are important factors of physical activity practice among adolescent population. Adolescents are priority targets for action against obesity and related comorbidities, and they should be more aware about the health benefits of physical activity practice. Programmes to promote physical activity and reductions in sedentary behaviour among not only adolescents but also their families, combined with a Mediterranean diet, would likely result in a better healthy profile in the future.

## Competing interests

The authors declare that they have no competing interests.

## Authors’ contributions

MMB and JAT conceived, designed, devised and supervised the study, MMB and JAT collected and supervised the samples. MMB, AC and JAT analysed the data and wrote the manuscript. AP and JAT obtained funding. All authors read and approved the final manuscript.

## Pre-publication history

The pre-publication history for this paper can be accessed here:

http://www.biomedcentral.com/1471-2458/12/718/prepub
